# Influences of COVID-19 vaccination policy on students’ vaccine acceptance

**DOI:** 10.4102/hsag.v28i0.2265

**Published:** 2023-08-30

**Authors:** Thuli G. Mthembu, Samantha Harrison, Kauthar Botha, Jessica Britz, Brittney Katts, Michaela Millar, Zia Sulliman, Vutlhari Zitha

**Affiliations:** 1 Department of Occupational Therapy, Faculty of Community and Health Sciences, University of the Western Cape, Bellville, South Africa

**Keywords:** vaccine acceptance, vaccine hesitancy, COVID-19 pandemic, coronavirus, health science students, mandatory COVID-19 vaccination policy

## Abstract

**Background:**

Higher education institutions (HEIs) developed and implemented a mandatory COVID-19 vaccination policy to facilitate vaccine acceptance and vaccination among universities’ staff and students. However, little is known about influences of the mandatory vaccination policy on health science students at a university and they tend to result in vaccine hesitancy.

**Aim:**

To explore the influences of the mandatory COVID-19 vaccine policy on health sciences students’ vaccine acceptance at HEIs in South Africa.

**Setting:**

The study was conducted in one of the universities in the Western Cape Province, South Africa.

**Methods:**

An interpretive qualitative exploratory-descriptive research was conducted with 10 participants who were selected using the purposive sampling method to participate in semi-structured interviews. Data were audio-recorded, transcribed verbatim and thematically analysed.

**Results:**

Two themes and 12 sub-themes were identified during the data analysis, namely individual and group influencing factors, as well as contextual influencing factors.

**Conclusion:**

This study revealed that the COVID-19 vaccination mandatory policy influenced the students’ quality of life, academic performance and well-being. The findings from this study indicate that there were perceived barriers related to personal and contextual influencing factors than benefits of COVID-19 vaccination.

**Contribution:**

The understanding of and insight into the influences of the mandatory vaccination policy provided a basis for further strategies that may be developed to address COVID-19 vaccine infodemic, vaccine hesitancy and its risk effects. This can be done through collaboration with different stakeholders to educate health science students about the perceived benefits of COVID-19 vaccination.

## Introduction

Understanding the complexity of a mandatory COVID-19 vaccination policy is vitally important, as it has received increased attention across a number of sectors including workplaces, school-based, higher education institutions (HEIs) and healthcare (Bardosh et al. 2022; Geng et al. 2022). The mandatory COVID-19 vaccination policy was built as a public health intervention strategy to address the debilitating COVID-19 pandemic by decreasing morbidity and mortality burdens (Savulescu 2021). Despite its clinical success, the mandatory COVID-19 vaccination policy has a number of problems related to accessibility-related restrictions and trust in government and scientific institutions (Schmelz & Bowles 2021, 2022). It was reported that people were restricted to access work, education, public transport and social life because of their COVID-19 vaccination status (Schmelz & Bowles 2021). These restrictions were perceived as barriers that perpetuated health and economic inequalities, infringement of human rights, stigma and social injustices (Bardosh et al. 2022). The accessibility-related restrictions have been found to disrupt the learning and teaching activities and the social structure of the population (Shindjabuluka, Ashipala & Likando 2022).

The mandatory COVID-19 vaccination policy was implemented to reduce the risk of transmission of the virus to other people (World Health Organization [WHO] 2022a) and to control the severe acute respiratory syndrome (Leask et al. 2021). In South African HEIs, mandatory COVID-19 vaccination policy was implemented to fast-track vaccine uptake among academic and professional workers, and students (Makhafola 2021; Moodley 2022; Potgieter et al. 2022). However, recent studies highlighted that the mandated vaccination does not optimise social responsibility and contract; instead, it promotes punitive, discriminatory, segregation and stigmatisation, which fuels the fire of vaccine hesitancy (Geng et al. 2022). Therefore, vaccine hesitancy becomes a complex and social behaviour that influences rollout and uptake of the vaccines (Cooper, Van Rooyen & Wiysonge 2021; Potgieter et al. 2022). Vaccine hesitancy refers to ‘delay in acceptance or refusal of vaccination despite availability of vaccination services’ (MacDonald & SAGE Working Group on Vaccine Hesitancy 2015:4163). Along with this vaccine hesitancy in HEIs, however, there is increasing concern over the determinants that result in vaccine decision-making to accept, delay or reject vaccines (MacDonald & SAGE Working Group on Vaccine Hesitancy 2015). Three categories of factors that influence vaccine hesitancy include contextual, individual and group, and vaccine or vaccination-specific influences (MacDonald & SAGE Working Group on Vaccine Hesitancy 2015).

Universities have been identified as high-risk areas for COVID-19 pandemic transmission because of overcrowding of students and staff during learning and teaching activities (Moodley 2022). In Geng et al.’s (2022) meta-analysis that investigated vaccine uptake and its influencing factors among students from medicine, dentistry and nursing, it was found that medical students had higher acceptance rate compared to dental students. This is further buttressed by Potgieter et al.’s (2022) quantitative analytical cross-sectional study that highlighted that vaccine hesitancy was high among dental students. Safety and efficacy of the vaccine was identified as drivers of vaccine hesitancy among the dental students (Potgieter et al. 2022; Cooper et al. 2021).

Health promotion is a process of enabling people to increase control over and improve their health through engagement in healthy behaviours to enhance the quality of life and well-being (Kumar & Preetha 2012). However, COVID-19 infodemic affected people’s control, as it continues to disseminate misconceptions, misinformation and conspiracy theories about the vaccine through social media (Bam 2022; Geng et al. 2022). For instance, research confirmed that COVID-19 infodemic perpetuated a risk of vaccine hesitancy and refusal of mandated vaccination among students, which influenced their participation in learning and teaching activities (Bam 2022; Geng et al. 2022; Potgieter et al. 2022). Nonetheless, mandated vaccine policies were not only implemented as a health promotion strategy to control the risk to spread the COVID-19 but it also exacerbated social anxieties and frustrations (Schmelz & Bowles 2021). However, it is unclear how the policies were formulated, which raised concerns among the population because there was a lack of clarity and transparency about the clumpy policy processes that were broken (McKee & Van Schalkwyk 2022). Therefore, Bardosh et al. (2022) suggest that empowering strategies for COVID-19 vaccination should be developed based on trust and public consultation to promote good health and well-being (i.e. goal 3).

The Health Belief Model (HBM) is increasingly recognised as a theoretical framework underpinning the understanding of and insight into the behaviour changes related to COVID-19 vaccination and willingness (Guidry et al. 2021; Wong et al. 2020; Zampetakis & Melas 2021). The HBM consists of six constructs: perceived susceptibility, perceived severity, perceived benefits, perceived barriers, self-efficacy to engage in a behaviour and cues to action (Wong et al. 2022). Perceived susceptibility is the belief that an individual has a high risk of being infected with the disease, which highlights the vulnerability of transmitting the virus to others (Wong et al. 2020; Zampetakis & Melas 2021). In this case, university students were reported to be a vulnerable population (Bourne et al. 2021; Moodley 2022). In accordance with perceived severity, it is a belief that the consequences of being infected with COVID-19 would be serious for the self and others (Zampetakis & Melas 2021). For instance, university students were perceived as a vulnerable population at a high risk of COVID-19 because of their engagement in learning activities in indoor lecture venues and living in residences (Bourne et al. 2021; Moodley 2022; Taye et al. 2021). Regarding perceived benefits, it is considered as individuals’ beliefs about the effectiveness of a variety of actions available to address the threat of the disease, such as COVID-19 vaccine (Wong et al. 2020). This is supported by the public health perspective that vaccination is part of the health management actions to reduce the risk of COVID-19 transmission among families, friends and relatives (Mose et al. 2022). However, Wong et al. (2020) cautioned about the perceived barriers, which are described as the belief that being vaccinated is restricted because of influences related to psychosocial, physical or financial factors. For instance, researchers found that influencing factors, such as religious beliefs, fear of side effects, misinformation and other practices played a significant role to reduce levels of vaccine uptake, adoption and acceptance (Bourne et al. 2021; Tolia et al. 2022; Uzochukwu et al. 2021). These influencing factors resulted in a vaccine hesitancy that posed the greatest challenges and alarming concern in the uptake of the COVID-19 vaccines among university students, which requires further investigation (Bourne et al. 2021; Potgieter et al. 2022; Uzochukwu et al. 2021). Regarding the cue of action in HBM, it involves the stimulus used to trigger people’s decision-making process to accept vaccination as a recommended health action (Wong et al. 2020). In addressing vaccine hesitancy, universities worldwide opted to implement a mandatory COVID-19 vaccination, as an external cue action supported to create a safe and caring environment for work and learning (Moodley 2022; Bourne et al. 2021; Makhafola 2021). In HBM, self-efficacy refers to the level of a person’s confidence to engage in healthy behaviour, such as vaccination against COVID-19. Students in Nevada HEIs shared their reasons for eagerness to vaccinate, which include a ‘desire to protect themselves, their friends and family members (88.7%, *n* = 1173), and to help get things back to normal (78.9%, *n* = 1044), (Elliott & Yang 2022:06). It was further highlighted that reluctance to COVID-19 vaccination among university students because of the potential negative side effects posed by the vaccine was identified as a concern (Elliott & Yang 2022).

Geng et al.’s (2022) meta-analysis review suggests that more research is needed on vaccination acceptance and willingness among universities’ students, as they are at higher risk of being infected. Much of the research on vaccine hesitancy up to now has been quantitative surveys in nature (Chamon et al. 2022; Cooper et al. 2021). Nevertheless, there is limited qualitative research that has been conducted about the effectiveness of the mandatory COVID-19 vaccination policy to increase vaccine acceptance rate among students at a university in the Western Cape Province. It is envisaged that scientific evidence from this research could be used to help universities, policy-makers, and government and non-government organisations to contribute to intervention strategies needed to address vaccine hesitancy. Therefore, this study aims to gain an understanding of and insight into how a mandatory COVID-19 vaccination policy influenced vaccine acceptance among health science students.

## Research purpose and objectives

The purpose of the study was to explore the influences of the COVID-19 vaccine policy on health sciences students’ vaccine acceptance at HEIs in South Africa. In accordance with the purpose of the study, several objectives were formulated including to:

- Explore the individual and group influencing factors of the COVID-19 vaccine policy on vaccination acceptance.
- Explore the contextual influencing factors of the COVID-19 vaccine policy on vaccine acceptance.

### Research methodology and design

An interpretive qualitative exploratory-descriptive research was used to conduct the study (Hunter, McCallum & Howes 2019). The focus of the research was to gain an understanding of and insight into vaccine acceptance and mandatory COVID-19 vaccination policy as a socially constructed phenomenon. Exploratory-descriptive research was used to provide multiple realities that were important to the students and highlight a clear picture of the situation.

### Population, sample and setting

The study occurred in one of the universities in the Western Cape Province, South Africa. It was conducted in the Faculty of Community and Health Sciences (CHS), which houses different Schools (Nursing and Public Health) and Departments (Psychology, Physiotherapy, Social Work, Dietetics, Occupational Therapy and Natural Medicine) where participants were recruited. In this faculty, the mandatory COVID-19 vaccination policy was implemented because it trains health professionals. Therefore, students had to be vaccinated so that they could provide services to the clients in clinical placements. Purposive sampling method was employed to select 10 participants to obtain in-depth and rich data (Campbell et al. 2020). Regarding the inclusion criteria, participants who were vaccinated and unvaccinated students between the ages of 18 and 45 had to be registered for the 2022 academic year within the CHS faculty at the university.

### Data collection

Semi-structured interviews were conducted between August and September 2022 to collect data. Semi-structured interviews have been proven both versatile and flexible as they can be combined with an individual and group interview method (Naz, Gulab & Aslam 2022). The semi-structured interviews were conducted using an interview guide (Kallio et al. 2016). The interview guide was structured into two sections: (1) introduction focusing on the participants’ demographic information and (2) possible questions related to COVID-19 vaccination policy, COVID-19 vaccination acceptance and influencing factors related to implementation of COVID-19 vaccination policy were developed based on the reviewed literature centred around the study and evaluated by the team, as suggested in Naz et al. (2022) and Kallio et al. (2016). The semi-structured interviews were conducted one-on-one (a researcher and a participant) in a digital platform such as Google Meet or Zoom, as a risk reduction measure to reduce COVID-19 transmission and lasted a duration between 15 min and 40 min. All the interviews were audio-recorded with the participants’ consent and transcribed verbatim to capture the participants’ responses. Digital data collected was downloaded and transferred onto a password-protected flash drive for security and safekeeping for 5 years. All data were de-identified and deleted from Zoom and Google Meet.

### Data analysis

A six-step method of thematic analysis was used and done manually in this study as described in Braun and Clark (2006). The six-step method of data analysis consisted of: (1) familiarisation with the data through reading and re-reading, (2) generating initial codes, (3) searching for themes, (4) reviewing themes, (5) defining themes and (6) writing-up of an interim report. All the steps were conducted under the supervision of the first author, which led to discussions about the themes and a consensus was reached. The research questions, aim and objectives of the study were used to guide the themes and sub-themes identified during data analysis. In enhancing validity of qualitative data analysis, the authors used reduction tables and searched literature in preparation for the discussion of findings (Cloutier & Ravasi 2021).

### Measures of rigour and trustworthiness

The study used the primary and secondary criteria to test for reliability and validity (Cypress 2017). Trustworthiness was enhanced through the primary criteria of credibility, transferability and confirmability (Klopper 2008). Prolonged engagement was used during data collection as authors spend enough time with the participants. Triangulation of multiple data sources was used to enhance credibility, as participants were recruited from different departments and selected using a purposive sampling method. Peer debriefing was used to enhance the credibility of the study through the discussions with the supervisor who has more knowledge about qualitative research. Through peer reviews, the authors sought advice and feedback throughout the research process and agreements were obtained on the identified themes from the data analysis (Green & Park 2021). Transferability was enhanced through a thick and accurate description of the research methodology used in this study. Confirmability audits were used to question every step taken to conduct and keep track, which enhanced the transparency and accuracy of qualitative data (Green & Park 2021). Reflexivity was ensured as the authors had to adhere to their ethical responsibilities, which involved reflecting on their roles, motives, biases and influences they brought to the research related to being vulnerable to COVID-19 pandemic, mandatory COVID-19 vaccination policy and vaccine acceptance at HEIs (Green & Park 2021). Secondary criteria of explicitness, creativity, thoroughness, congruency and sensitivity contributed to the test of validity (Whittemore, Chase & Mendle 2001). The results are presented in an explicit manner by considering the context and phenomenon of the study. Hence, the data were organised, presented and analysed in a credible way. Thoroughness was ensured by the connection between the themes, aims and research questions, which enhanced congruence of the research methods, current study, existing research and findings. Sensitivity enabled authors to contribute to validity by respecting participants and their human dignity, which strengthened ethical considerations.

### Ethical considerations

Ethical clearance of the study was sought from the University of the Western Cape Humanities and Social Sciences Research Ethics Committee (HSSREC) and reference number: HS22/5/26. The research committee at the Faculty of Community and Health Sciences approved the study. All the participants were informed about the study and they gave informed consent. Participants were informed that their participation was voluntary and they could withdraw from the study without any repercussion. Anonymity was enhanced with numbers to de-identify the participants, as part of the protection of personal information in line with the *POPI Act 2013*. All data are stored in a safe place and will be discarded according to the university’s data management policy.

### Findings

Ten participants registered as students at the four departments (i.e., dietetics, occupational therapy, physiotherapy and social work) of the Faculty of Community and Health Sciences consented to be part of the study. Table 1 presents the participants’ characteristics and they were between the ages of 22 and 41 years, with an average mean and standard deviation of 24 ± 5.85. It is well established from a variety of studies that gender is one of the characteristics that influences willingness to vaccinate and support COVID-19 mandatory policy (Attwell, Roberts & Ji 2023). These studies indicate that women were more likely to accept COVID-19 vaccination compared to men. However, the findings of this study differ from the published work because the female participants opposed the implementation of the COVID-19 mandatory policy. It is apparent from Table 1 that only female participants were part of the study, and these findings suggest that there is a need for study with male participants.

**TABLE 1:** Characteristics of the participants (*N* = 10).

Characteristics	Total
**Age†**	
21–30 years	9
31–40 years	0
41–50 years	1
**Gender**	
Male	0
Female	10
**Departments**	
Dietetics	1
Occupational therapy	6
Physiotherapy	1
Social work	2
**Year level of study**	
Year 1	0
Year 2	0
Year 3	1
Year 4	9

*Source:* Botha, K., Britz, J., Harrison, S., Katts, B., Miller, M., Sulliman, Z. et al., 2022, ‘The influences of the COVID-19 vaccination policy on the students’ vaccine acceptance at a higher education institution in South Africa’, Undergraduate dissertation, University of the Western Cape, Cape Town

s.d., standard deviation.

† , s.d. 24 ± 5.85.

## Presentation of the themes and sub-themes integrated with their discussion

Two themes and 12 sub-themes were identified during the data analysis namely: (1) individual and group influencing factors and (2) contextual influencing factors (see Table 2). These themes and sub-themes are presented and discussed in conjunction with corresponding and conflicting literatures, and the HBM.

**TABLE 2:** Themes and sub-themes.

Themes	Sub-themes
Individual and group influencing factors	Restricted autonomy and access to learning facilities Students’ academic performance Students’ values and belief systems Mandated vaccination policy influenced mental health Deprived vaccine exemptions
Contextual influencing factors	COVID-19 vaccine-specific issues COVID-19 vaccination certificate and status COVID-19 vaccine infodemic COVID-19 vaccine bullying in social media Restriction to social participation Unvaccinated students’ reasonable accommodation Discontinuation of the mandatory COVID-19 vaccination policy

*Source:* Botha, K., Britz, J., Harrison, S., Katts, B., Miller, M., Sulliman, Z. et al., 2022, ‘The influences of the COVID-19 vaccination policy on the students’ vaccine acceptance at a higher education institution in South Africa’, Undergraduate dissertation, University of the Western Cape, Cape Town

### Theme 1: Individual and group influencing factors

The first theme deals with the individual and group factors that influenced vaccine acceptance, as expected from the mandatory COVID-19 vaccination policy. In accordance with the analysed data, the following five sub-themes emerged, namely, restricted autonomy and access to learning facilities, students’ values and belief system, students’ academic performance, the vaccination policy influenced students’ mental health and students’ deprived vaccine exemptions.

#### Sub-theme 1: Restricted autonomy and access to learning facilities

The participants highlighted that mandatory COVID-19 vaccination policy infringed their ethical right of autonomy because they were not given an opportunity to make informed decisions about vaccine uptake. Furthermore, the participants felt that they were obligated to be vaccinated as a requirement to continue with their studies:

‘I think it is not fair that students are basically ‘forced’ to get the vaccine, and if they do not, they will be denied access to learning facilities.’ (Participant 9, Physiotherapy Student, 23 years old)

‘I think it is a really reliable and safer measure of protecting ourselves from the virus. I, however, do not agree with how it was enforced, especially for individuals in the healthcare environment. I do believe in autonomy, and every person has the right to decide what goes in their bodies.’ (Participant 8, Social Work Student, 24 years old)

These findings are consistent with Chamon et al. (2022) who highlighted that COVID-19 vaccine policies are contrary to liberal values of bodily autonomy, freedom of choice, human rights and informed consent. Hence, one participant reported that universities’ mandatory COVID-19 vaccination policies were contradictory with the South African government:

‘It wasn’t mandatory for everyone in the country to get vaccinated but for the university to go and say, look here no student left behind, but then at the same time, say that you need to be vaccinated to complete your degree or further your studies. That obviously made me very upset … my options left me with either being vaccinated or not.’ (Participant 1, Social Work Student, 22 years old)

These findings raised intriguing questions regarding the nature and extent of how mandatory COVID-19 vaccination policies were implemented at universities. In explaining the rationale behind the mandated vaccination, Moodley (2022) asserts that both the South African Bill of Rights and public health ethics approach permit limitation of human rights if it is justifiable in accordance with the purpose of promoting human dignity, equality, freedom, good living and solidarity.

#### Sub-theme 2: Students’ values and belief systems

Participants expressed that they were unfairly treated by the universities that adopted the mandatory COVID-19 vaccination policy, as it was against their values and belief systems. The participants shared a sense of discontentment because the universities’ mandatory vaccination seemed to have superseded the non-obligatory South Africa’s laws. However, participants shared feelings of disappointment because they were compelled to vaccinate so that they might proceed with their studies. In addition, the participants shared that they were caught between a rock and a hard place in vaccine decision-making because they had to choose between values, belief systems, studies and vaccination:

‘My religious beliefs, because I am Catholic, and we were told that they actually should not be taking the vaccination. And I had to put my religious beliefs and my personal beliefs to one side, because, you know, what am I going to do if I don’t get vaccinated and I’m not allowed back to university? Where do I go from there?’ (Participant 1, Social Work Student, 22 years old)

Comparison of the findings with those of other studies confirms that cultural and religious beliefs were contextual factors that influenced both vaccine hesitancy and acceptance (Bourne et al. 2021; Majee et al. 2022). However, the findings highlighted a particular relevant ethical dilemma related to values and belief systems, which facilitated professional agency among the students so that they can be responsible for their own learning and commit to their academic performance (Toom et al. 2021).

#### Sub-theme 3: Students’ academic performance

Participants shared that the negative influences related to home environment and online learning, which resulted in a poor academic performance and a sense of disheartened. The participants further indicated that online learning was not their preferred method and they had troubles:

‘Apart from being at home in an environment that is not very conducive for learning and having to learn online for the first time in my life. I also struggled; I was demotivated because it was not my preferred way of learning. So, I could actually see my marks on the stage.’ (Participant 1, Social Work Student, 22 years old)

The present findings are consistent with studies (Maatuk et al. 2022; Shindjabuluka et al. 2022) that highlighted that COVID-19 pandemic was an enabler that facilitated online learning and teaching activities using a variety of platforms. Nevertheless, Shindjabuluka et al. (2022) validated the findings of the current study, who reported the challenges that influenced the rapid migration to online learning, as students struggled with connectivity and laptops. It can therefore be suggested that universities should equip educators with learning and teaching strategies that may assist students to develop adaptive skills needed for online learning.

Participants accentuated that COVID-19 emergence mandated the migration to online learning platforms, which were incomparable with their preferred learning styles, such as auditory and practical learners. In addition to the negative influences of the migration, the participants mentioned that online learning restricted their exposure to practical and experiential learning:

‘We were kind of forced to, you know, go from face to face or physical lectures to learning online, which was especially difficult for me because I am an auditory learner.’ (Participant 4, Occupational Therapy Student, 22 years old)

‘Studying occupational therapy is a very practical degree and studying online and seeing videos of things that you should be experiencing in person was a big, big downfall.’ (Participant 10, Occupational Therapy Student, 25 years old)

These results differ from some published studies (Maatuk et al. 2022; Shindjabuluka et al. 2022). However, they are consistent with those of Brown, Cosgriff and French (2008) who reported that occupational therapy students are accommodators because their preferred learning style involves concrete experience, active experimentation, risk taking and they enjoy working with people. Some of the issues emerging from these findings relate specifically to the need to adapt and reform online learning and teaching strategies to accommodate the preferred learning styles of our students (Frantz, Roman & De Jager 2017).

#### Sub-theme 4: Mandated vaccination policy influenced students’ mental health

Participants shared that the COVID-19 mandated vaccination policy has had a negative influence on their mental health and academic performance:

‘It made my anxiety levels shoot through the roof, it made me very angry, I was very upset and I thought it was really unfair.’ (Participant 3, Occupational Therapy Student, 41 years old)

‘The chaos that came with the pandemic affected my mental health, which had a major impact on my studies.’ (Participant 9, Physiotherapy Student, 23 years old)

It is evident that the implementation of the mandatory COVID-19 vaccination policies evoked distressing feelings, which debilitated the participants’ mental health status. The findings of the present study were in resonant with Souliotis et al. (2022), who found that there was a positive association between COVID-19 vaccination and mental health. This finding has important implications for universities to strengthen the mental health programmes so that students’ mental health and well-being will be considered, as supported by goal 3 of the sustainable development goals.

#### Sub-theme 5: Students’ deprived vaccine exemptions

Participants reported that applying for vaccine exemptions because of medical and religious reasons was a worthless effort, as elucidated by a dietetics student: ‘I didn’t because it was just too much of an effort’ (Participant 6, Dietetics Student, 22 years old). The participants enunciated that the process of exemption from the vaccination was tedious, as there was no assurance that they were going to be exempted:

‘I know two individuals that did apply for an exemption and they did not get it, one on religious grounds and one on medical grounds. So, at that point, I thought to myself, look here, is it worth the fight?’ (Participant 1, Social Work Student, 22 years old)

‘After like two to three weeks of applying for the exemption, getting all my facts and laws together to apply for exemption. I was not able to enter fieldwork or like to visit fieldwork placements for the first week so I was at home. During the second week when my block had to start, I was pushed. I was denied exemption twice and my year rep said, unfortunately, she has to tell me that it’s either the vaccination or I cannot continue my degree, so I had to take it and I had to send proof the moment I was vaccinated.’ (Participant 2 Occupational Therapy Student, 23 years old)

The results of this study indicate that universities’ mandatory vaccination policies tightened the process of applying for a COVID-19 vaccine exemption based on medical, religious and personal grounds (Bardosh et al. 2022). However, the findings revealed that unvaccinated students experienced accessibility challenges related to resources, facilities and inability to register. As a result, the mandated vaccination has left unvaccinated students with a problematic situation, as they could not proceed with their studies, thus, delaying their ability to graduate (Bardosh et al. 2022).

### Theme 2: Contextual influencing factors

The second theme focuses on the contextual factors influencing vaccine acceptance during the use of the mandatory COVID-19 vaccination policy. According to the data that were thematically analysed, the following seven sub-themes emerged, namely, COVID-19 vaccine-specific issues, COVID-19 vaccination certificate and status, COVID-19 vaccine infodemic, COVID-19 vaccine bullying in social media, restricted social participation, unvaccinated students’ reasonable accommodation and discontinuation of the mandatory COVID-19 vaccination policy.

#### Sub-theme 1: COVID-19 vaccine-specific issues

The emergence of COVID-19 in 2020 triggered a huge amount of innovative scientific development and distribution of safe and effective COVID-19 vaccines as well as inquiry; however, it should be noted that the COVID-19 vaccine process was laden with many challenges (Forman et al. 2021). For instance, HEIs introduced a mandatory COVID-19 vaccination policy as a strategy to encourage the uptake of vaccines for the students and staff (Moodley 2022; Potgieter et al. 2022). In the present study, a lack of understanding of the COVID-19 vaccines was perceived as one of the challenges that led the participants to be reluctant to accept the mandatory COVID-19 vaccination. Participants expressed their concerns that COVID-19 vaccines were rapidly produced and rolled out with little information pertaining to the adverse effects, which resulted in vaccine hesitancy:

‘When the vaccination was initially rolled out, I was quite skeptical, mostly because it came about so fast, considering that COVID was like a relatively new thing. So that kind of influenced me to be vaccine hesitant. A lot of people are falling sick, or people dying of the side effects of the vaccination.’ (Participant 1, Social Work Student, 22 years old)

‘I don’t support it because I feel it wasn’t researched well enough and then it was forced on people and now, now negative effects are coming out.’ (Participant 3, Occupational Therapy Student, 41 years old)

A prominent reason that resulted in students’ vaccine hesitancy could be that there was less time allocated for the vaccine trial processes to establish its safety and efficacy before it was licensed, as highlighted in Moodley (2022) and Chamon et al. (2022). This explanation about the lack of understanding of and information about COVID-19 vaccine is consistent with that of Cooper et al. (2021) who highlighted that insufficient knowledge and mistrust towards vaccines tend to influence vaccination acceptance and coverage needed for herd immunity. As a result, HEIs should consider inclusive vaccination campaigns where students can be provided with a transparent knowledge about vaccine hesitancy through engagement in dialogue to address the students’ concerns.

#### Sub-theme 2: COVID-19 vaccination certificate and status

Participants stated that they understood the need for vaccination; however, they indicated that they were treated unfairly by the universities that imposed the COVID-19 vaccines upon everyone, which appeared as a requirement for employment and education that violated their rights:

‘Issuing the vaccine mandate in our country might have been a rational decision considering how fast the virus was spreading and the rise of death cases related to the virus. It, however, became unfair that it became a job requirement for employment and those students were only allowed access back to institutions once they provided a vaccination certificate.’ (Participant 8, Social Work Student, 24 years old)

Thus, the results from earlier studies corroborate with the present findings that demonstrated a strong and consistent ethical debates and concerns around the use of the vaccine passports as employer and university vaccine requirements to restrict access to work, education and public spaces (Chamon et al. 2022; Forman et al. 2021).

In prioritising the need of education, participants’ vaccine decision-making resulted in COVID-19 vaccine acceptance and vaccination. Therefore, the vaccination certificate and status were used as an access passport for class attendance, clinical and fieldwork placements, and residence. The vaccination decisions were guided by the mandated vaccine and exclusions:

‘We were told plain and simple that if we don’t get vaccinated, we aren’t allowed to return to face to face lectures, which is obviously very much crucial and that we wouldn’t be able to complete our practical’s which we need to graduate.’ (Participant 1, Social Work Student, 22 years old)

‘Obviously, my studies come first.’ (Participant 6, Dietetics Student, 22 years old)

‘Then, I got the Johnson & Johnson because that was the only vaccine where you only needed one shot and you could enter practical block whereas for the Pfizer, I think there was a period between the two vaccinations and during that period you were still not allowed to enter block so I would’ve basically missed, well failed my first block of fourth year.’ (Participant 2, Occupational Therapy Student, 23 years old)

Being unvaccinated was perceived as a barrier to engagement in learning and teaching activities as well as fieldwork. The participants expressed that the consequences of being unvaccinated were hard, as they would have missed the opportunity to complete their training:

‘I was excluded and I asked them if there would be like an online catch up and the university just said no, you missed out on this, there will be no catching you up on this, we will not have an online catch up on it or there was lectures happening after the placement visits and I was also not allowed to attend them, they did not make it online for us’ (Participant 2, Occupational Theraphy, Student, 22 years old)

The findings are congruent with Bardosh et al.’s (2022) argument that conditioning ‘access to health, work, travel and social activities on COVID-19 vaccination status is inherently punitive, discriminatory and coercive.’ It seems possible that these results are because of the fact that the university students were a vulnerable population that remained hesitant to vaccinate. Universities opted to move to mandated vaccination efforts with the aim of enabling students to return to campus life, as supported by the studies on mandatory COVID-19 vaccination policy that accentuated the need of the vaccine certification and status to monitor university students (Bourne et al. 2021; Chamon et al. 2022; Moodley 2022). The findings of the present study further corroborate with Bardosh et al. (2022) who reported that some people vaccinated to prevent refusal of access to livelihood.

In relation to the HBM, the vaccination status was not only perceived as a barrier that violated participants’ freedom; however, it was also perceived as a benefit of vaccination that facilitated return to a normal life and access to the university’s residence. These findings corroborated previous research reporting the importance of returning to normal life (Chamon et al. 2022):

‘For me to be able to get back to the campus residence, I had to vaccinate at first, which I was okay with. And that made it simpler for me to be able to be back and stay at the residence because I was vaccinated and those who are not vaccinated, they were not allowed at all.’ (Participant 4, Occupational Therapy Student, 22 years old)

#### Sub-theme 3: COVID-19 vaccine infodemic

One of the most significant current discussions in relation to the rollout and uptake of COVID-19 vaccines is COVID-19 infodemic (Bam 2022; Majee et al. 2022). The WHO (2022a,b) refers to COVID-19 infodemic as the spread of a large amount of fake news, rumours and information that mislead the public about emerging health issues and disease outbreaks in digital and social media. In relation to COVID-19 infodemic, participants expressed that misconceptions about COVID-19 vaccine were spread in social media in the form of pictures, incorrect information, word of mouth and texts resulting in vaccine hesitancy. It is evident that incorrect information emanating from the social media has increased the risk of mistrust about the vaccination against COVID-19 pandemic:

‘Pictures that have been posted by people that the vaccine causes you blisters.’ (Participant 7, Occupational Therapy Student, 22 years old)

‘Word of mouth has become such a big thing; the vaccine has had a negative connotation attached to it, which false information has spread. This has made a lot of people hesitant to get the vaccine … no one knows of the long-term health risks, and as women, if it would compromise our fertility.’ (Participant 7, Social Work Student, 24 years old)

In accordance with the COVID-19 vaccine infodemic, Moodley (2022) cautions about existing misperceptions that COVID-19 vaccine contains aborted cells, which were evident among the participants who shared their concerns as women at a child-bearing age that they were scared of vaccination. As a result, the participants’ vaccine decision-making was influenced by the fear of infertility:

‘I read a lot of social media posts on why the vaccine is bad for you in the sense of people that couldn’t be able to get pregnant … So yes, that had an influence on me. If there were people that actually were challenged with health issues after the vaccine. I think it just made me feel stronger about not having the vaccine. Even if it is just two out of ten people, who don’t have a good experience with the vaccine that does influence me.’ (Participant 2, Occupational Therapy Student, 23 years old)

The need to disseminate correct information about COVID-19 vaccine has been highlighted by Bam (2022) and Chamon et al. (2022), who suggested that citizens from all ages should be provided with positive scientific reviewed knowledge. However, the participants shared that the younger generation do not seek information from scientific researchers and health professionals to validate their understanding of the COVID-19 vaccine:

‘Today’s world revolves around social media. The opinion of celebrities and social media influencers receives immense support from individuals, especially the younger population. Individuals tend to not do their own research and gain knowledge about the vaccine from certified scientists and medical personnel in order to make a valid and appropriate decision that will benefit their health. Instead, they follow the opinions of celebrities and influencers with no scientific evidence.’ (Participant 9, Physiotherapy Student, 23 years old)

It is evident from the findings that COVID-19 infodemic disseminated misconceptions and misleading news about COVID-19 vaccine, which is in line with Potgieter et al. (2022) and Ahmed (2022) who highlighted the influences of the communication and media environment. Therefore, the findings of this study suggest that HEIs should consider investing in an infodemic management that promotes collaboration with other stakeholders such as medical health practitioners, community leaders, religious leaders and certified scientists to educate the students about the COVID-19 vaccines and its risk, and build resilience to misinformation (Bam 2022; Lelisho et al. 2022; WHO 2022a,b).

#### Sub-theme 4: COVID-19 vaccine bullying in social media

When the participants were asked about the influences of communication and social media on vaccination acceptance, participants indicated that they experienced COVID-19 vaccine bullying in social media, which appeared as a pushing force towards vaccine acceptance and vaccination. The participants shared that there were online repercussions for delayed vaccination:

‘You know, it was like all over the news, you know, and that you need to get vaccinated. On Instagram, everyone was like being vaccinated. And if you were not vaccinated, then people online would backlash you for not being vaccinated.’ (Participant 5, Occupational Therapy Student, 22 years old)

In contrast to the findings reported in this sub-theme, Foley and Gurakar (2022) revealed that bullying tends to be more prominent among anti-vaccine and vaccine-hesitant users, who perceive it as a social sanction.

#### Sub-theme 5: Restricted social participation

Participants expressed how the unvaccinated status restricted their social participation and rugby fields as part of collective actions:

‘I wasn’t able to get on the campus. So, then the rugby games that I would go to normally, before COVID-19 pandemic. We weren’t able to go and see that because we weren’t vaccinated … when you feel left out, like that’s playing with your emotions, like, oh, my gosh, I see all the videos of my friends going, but I’m not able to go.’ (Participant 6, Dietetics Student, 22 years old)

In relation to the HBM, perceived barriers were of concern that led the participants to be more worried about their social participation than perceived severity of being infected with COVID-19 (Chamon et al. 2022). Consequently, these findings echo Bardosh et al. (2022) who highlighted that mandated COVID-19 vaccination policies perpetuated social anxieties and anger of those who were unvaccinated. Consequently, unvaccinated students felt discriminated against and segregated from their friends, as they were denied access to engage in sports and social life (Bardosh et al. 2022).

#### Sub-theme 6: Unvaccinated students’ reasonable accommodation

Participants expressed that they felt their constitutional rights and autonomy were infringed by the mandatory COVID-19 vaccination policy that was implemented at universities. However, the participants felt that the universities should have explored other risk reduction strategies to accommodate those who were unvaccinated and who cannot be immunised to attend classes:

‘I understand that some of the clinical placements wanted us also to be vaccinated but if there were placements that did not need or did not want to see vaccination cards. I think the university could have at least placed us at those placements rather than to have us forced to get the vaccinations.’ (Participant 2, Occupational Therapy Student, 23 years old)

In response to the reasonable accommodation measures, Bardosh et al. (2022) argue that weekly negative COVID-19 tests could result in financial burden for unvaccinated people.

#### Sub-theme 7: Discontinuation of the mandatory COVID-19 vaccination policy

Participants expressed that the discontinuation of the mandatory COVID-19 vaccination policy at the universities brought a sense of normality:

‘I think because they scrubbed the vaccination mandate, as the government is no longer urging people to get vaccinated, but we are going back to some sense of normality. What was the point in forcing everyone?’ (Participant 1, Social Work Student, 22 years old)

Based on the lens of HBM, the findings highlighted that termination of the cue of actions of the mandatory COVID-19 policy resulted in the perceived benefits of returning to a normal life without considering the severity of being infected with COVID-19. These findings of normal life resonate with a call for decolonial love and its ethics of liberation for all humans to live together harmoniously after the termination of the COVID-19 mandatory vaccine policies (Ndlovu-Gatsheni 2020). The return to normal life forms part of the perceived benefits of transitioning from online learning to face-to-face classes and ameliorates a ubiquitous gap between privileged and historically disadvantaged students at universities (Moodley 2022). Consequently, university students’ academic performance would be enhanced to achieve the quality of education, as stipulated in goal 4 of the Agenda 2030 of sustainable development goals.

## Limitations and recommendations

A limitation of this study is that it was conducted with a small size of female students only registered in one faculty at a university, which might have omitted significant perspectives of male and female students from faculties other than Community and Health Sciences. However, an implication of these findings is that both COVID-19 vaccine hesitancy and vaccine acceptance should be considered when revising the mandatory COVID-19 vaccination policy for HEIs. Therefore, a number of important recommendations related to amendment of the mandatory COVID-19 vaccination policy, Faculty of Community and Health Sciences, learning and teaching strategies, and future research are developed based on the findings and literature in the field of vaccine hesitancy.

Regarding the amendment of the mandatory COVID-19 vaccination policy, vaccine infodemic management should be incorporated so that all stakeholders will be upskilled with knowledge and skills of how to convey correct information. Additionally, the policy should provide guidelines for balancing the South African Bill of Rights (section 36) and public health ethic approach to address limitation of rights for the sake of human dignity, public good, solidarity, equality and freedom (Moodley 2022).

There is, therefore, a definite need for the Faculties of Community and Health Sciences to play a vital role in conscientising university staff, students, visitors and the public as a whole about COVID-19 vaccination, as part of social impact. Through adopting quality education (goal 4) about COVID-19 vaccination to promote the good health and well-being (goal 3) of the public masses, this could be done by ensuring that people contribute to healthy cities and communities (goal 11) with collaborative partnership (goal 17). These efforts could be integrated in the community engagement and inclusive vaccination campaigns with the involvement of governments, the media, health professionals, law enforcement officers, community leaders, academics and others (Bam 2022; Forman et al. 2021).

In relation to learning and teaching strategies needed for fostering quality education at universities during pandemics and disasters, the findings from the present study highlighted that students had preferred learning styles, which were not accommodated in an online learning and teaching platform. Therefore, continued efforts are needed to make campuses more accessible to allow university students to complete ‘Kolb Learning Style Inventory (LSI) and VARK Questionnaire during the first semester of one of their courses’ as suggested by Brown et al. (2008). This information can be used to develop targeted interventions aimed at strengthening learning modes needed to build resilience among university students to cope with the challenges identified in the current study.

In planning for future research, it would be interesting to use mixed methods research designs to compare experiences of both female and male university students and staff regarding COVID-19 vaccination and its influencing factors to their health and well-being, and quality of education from different faculties. This kind of study will add value to the body of knowledge of vaccine hesitancy, as previous studies highlighted that university students tend to be reluctant to accept vaccination (Bourne et al. 2021; Moodley 2022; Potgieter et al. 2022).

## Conclusion

Returning to the purpose of this study, it is now possible to state that the influences of the mandatory COVID-19 vaccine policy on university students’ vaccine acceptance were explored. The findings indicated that the perceived barriers to vaccine acceptance could be ameliorated through community campaigns related to COVID-19 vaccination and its risk effects, and vaccine hesitancy through collaboration with different stakeholders. One of the more significant findings to emerge from this study is that COVID-19 vaccination infodemic should be addressed to reduce disinformation and misinformation spread in a variety of platforms. The present study adds to the growing body of research that indicates a need to deal with the influencing factors of mandated vaccination.
